# WHAT WORKS AND WHAT IS MISSING: A PRECEPTOR AND AGENCY PERSPECTIVE

**DOI:** 10.1093/geroni/igad104.0489

**Published:** 2023-12-21

**Authors:** Phyllis Greenberg, Jodi Danielson

**Affiliations:** St. Cloud State University, St. Cloud, Minnesota, United States; Good Shepherd Community, Sauk Rapids, Minnesota, United States

## Abstract

As has been discussed, the population of older adults is growing at a rapid pace and the need for staff in residential environments has grown to a crisis situation. Agencies are in dire need of staff at all levels from CNAs to nurses, social workers and administrators. There is an opportunity for agencies to help train the new generation of age savvy staff by working with universities to provide hands-on experience such as internships, practicum, and field work. This presentation examines the preceptor and community agency perspective of working with students interested in aging. We explore how to achieve a win/win/win/win experience (student, preceptor-agency/university/older adult). The presenters represent a agency preceptor and a preceptor-faculty member. Key areas for consideration include: 1) Experience/education of students; 2) Time commitment for preceptors/ agencies; 3) Agency and university support for preceptors; 4) Support for students from preceptors, agencies and the university; 5) Flexibility in responsibilities and scheduling between agencies, preceptors, and students; and 6) Ensuring that the experience is valuable to both students and agencies. Presenters will discuss strategies for meeting the needs of students and agencies while at the same time meeting the requirements of the university. These strategies include student involvement in day-to-day activities, as well as projects, teaching moments, providing support to preceptors as well as on-going communication and feedback. The goal is to provide an environment (both agency and university) that fosters growing an age savvy workforce.

